# Monkeypox outbreak in the age of COVID-19: a new global health emergency

**DOI:** 10.1186/s40779-022-00419-7

**Published:** 2022-10-02

**Authors:** Rahim Hirani, Dawood Rashid, Joshua Lewis, Rasheed Hosein-Woodley, Ali Issani

**Affiliations:** 1 New York Medical College School of Medicine, 1501 Old Farm Road, Valhalla, NY 10595 USA; 2 Department of Emergency Medicine, Aga Khan University, Karachi, 74800 Pakistan

**Keywords:** Monkeypox virus, Coronavirus disease 2019 (COVID-19), Global health

Dear Editor,

The World Health Organization recently declared monkeypox as a global emergency after reporting more than 57,000 new cases worldwide [1]. Monkeypox is an orthopoxvirus similar in nature to the Variola virus, which is a causative agent for smallpox [2]. Monkeypox virus presents as a unique challenge for global health and should be regarded with grave concern as the vast majority of cases are occurring in countries where the disease is not considered endemic. Furthermore, the spread of this pathogen is occurring concomitantly as the world is still engaged in a battle with the coronavirus disease 2019 (COVID-19) pandemic, which has caused substantial damage to global healthcare infrastructure. If monkeypox continues its rapid spread, hospitals could be quickly overwhelmed by both COVID-19 and monkeypox cases. Now more than ever, early symptom recognition and use of all available treatments to contain monkeypox outbreaks are vital. Early precautions including early recognition of symptoms by physicians, use of currently available treatments, and promoting precautionary measures in at-risk populations may be vital in preventing hospital burden and further physician burnout.

The effects of COVID-19 have reverberated throughout a multitude of communities across the world. People have been profoundly affected, especially that of front-line workers such as clinicians. A surge in emergency room visits coupled with a disease that at the time had no real cure, substantially increased the workload on healthcare workers [3]. This led to a surge in burnout and mental health conditions due to increased stress, depression, and anxiety. Likewise, it also caused an increase in suicide rates amongst physicians. While their services were the need of the hour, especially during the peak of the pandemic, their own health could have affected how they treat their patients, resulting in unintentional suboptimal care. We argue that this recent monkeypox outbreak could exacerbate these effects and further overwhelm our healthcare workers if precautions are not taken in time.

Whilst combating the psychological burden associated with the treatment of diseases such as COVID-19 and monkeypox poses a systemic challenge for institutions and will likely be a long-term effort—the clinical component also presents a uniquely challenging perspective. Currently, no standard treatment exists for management of monkeypox infections. Clinical trials are being created to test the efficacy of the antiviral drug tecovirimat in clade 3 monkeypox infections in humans. Tecovirimat has previously been approved in accordance with the Food and Drug Administration (FDA) Animal Rule for treatment of smallpox, by using non-human primates infected with monkeypox as a model for smallpox [4]. As the only FDA approved treatment for smallpox, the Centers for Disease Control and Prevention (CDC) has allowed clinical use of tecovirimat under expanded access protocol. Although animal models do not always confer efficacy in humans, three early case studies in Massachusetts, using 600 mg tecovirimat twice daily, have shown promising early results. In all three cases, there was symptom improvement after initiation of tecovirimat therapy with two cases showing a halt in progression of skin lesions within 2–4 d of treatment [5]. A retrospective cohort study done in the UK by Adler et al. [6] for the cases between 2018 and 2021 that examined both tecovirimat (600 mg twice daily) and brincidofovir (200 mg weekly for three weeks) had similar findings. One patient treated with 600 mg tecovirimat twice daily developed no new lesions 24 h after initiation of therapy with blood and upper respiratory samples being polymerase chain reaction (PCR) negative after 48 h. In the three patients treated with brincidofovir, there was some reduction in monkeypox viral PCR cycle threshold, but improvements were not consistent between the three patients. In addition, all three patients developed elevated alanine transaminase levels and therapy was discontinued early. Although available data is currently limited, early case studies demonstrate the efficacy and safety of tecovirimat as the best available treatment for monkeypox. Because it will take time for clinical trials for tecovirimat to be approved and completed, treatment under the CDC expanded access protocol may be vital for containing the virus and preventing further burden on global healthcare systems.

Furthermore, the CDC recommends two vaccination protocols for gaining immunization towards monkeypox. In 2015, the ACAM2000 vaccine was approved for immunization towards orthopoxvirus, specifically that of smallpox. The ACAM2000 is a live, attenuated virus that carries vaccinia virus which belongs to the same family of viruses such as monkeypox. However, in 2021 it was voted by the Advisory Committee on Immunization Practices that the JYNNEOS vaccine offers greater pre-exposure protection than the ACAM2000 vaccination and as such should be used as the gold standard for treatment for the foreseeable future. Additionally, it has been shown that the ACAM2000 vaccine can lead to greater side-effects compared with JYNNEOS vaccine especially in populations who are immunocompromised [7]. With the availability of two vaccines on the market, it is imperative to engage in public health projects to encourage groups who are at high-risk for exposure to monkeypox to procure either of these vaccines.

With the rapid rise in cases of monkeypox, it is crucial that we utilize an active mode of pedagogy. We must utilize the newfound knowledge to develop early symptom recognition and treatment strategies, as well as promoting awareness in at-risk populations, which would allow us to combat this new threat as a community. We must work collectively in identifying issues that can be exacerbated by the onset of another pandemic and develop an approach to mitigate those issues.

## Data Availability

Not applicable.
